# Entrepreneurial Intention and Perceived Social Support From Academics-Scientists at Chilean Universities

**DOI:** 10.3389/fpsyg.2021.682632

**Published:** 2021-07-07

**Authors:** Eduardo Acuña-Duran, Daniela Pradenas-Wilson, Juan Carlos Oyanedel, Roberto Jalon-Gardella

**Affiliations:** ^1^Facultad de Economía y Negocios, Universidad Andres Bello, Santiago, Chile; ^2^Doctorado en Psicología, UCA Pontificia Universidad Católica Argentina, Buenos Aires, Argentina; ^3^Facultad de Educación y Ciencias Sociales, Universidad Andres Bello, Santiago, Chile

**Keywords:** academic entrepreneurship, Planned Behavior Theory, structural equation modeling, academic entrepreneurial intention, entrepreneurial ecosystem

## Abstract

Within Ajzen's Planned Behavior Theory framework, this article tests a model to estimate the predictors of entrepreneurial intention in academic scientists working in Chile. We adapted into Spanish the entrepreneurship intention questionnaire. We tested the entrepreneurship intention model on a sample of 1,027 scientists leading research projects funded by the Chilean Scientific and Technological Development Fund (FONDECYT), the country's primary scientific research grant. The results show strong empirical support for the entrepreneurship intention model proposed while highlighting some critical issues specific to entrepreneurial intention in scientists. In particular, we found an indirect effect of perceived subjective social support on entrepreneurial intention, which is mediated by entrepreneurial attitude and perceived behavioral control toward entrepreneurship. These results suggest that policies orientated toward promoting academic entrepreneurship should include developing a healthy social environment toward it, meaning that entrepreneurial intention is not only an individual but an organizational challenge. These policies should analyze the social norms guiding the scientists' reference groups to increase their effectiveness.

## Introduction

Research on scientists' entrepreneurial activity has identified social and institutional factors that affect it (Kenney and Richard Goe, 2004; Fini et al., 2009; Nosella and Grimaldi, 2009). Nonetheless, research is still scarce about analyzing individual scientists as relevant actors in the academic entrepreneurship process (from now on, AE). Addressing the reasons for individuals, particularly academic-scientists, in carrying out entrepreneurship initiatives is a central issue. Understanding these phenomena can better manage the development of more appropriate policies to promote AE. To understand AE it is crucial to know how an academic scientist becomes an entrepreneur and which factors make entrepreneurship an attractive option.

Research has focused on the study of the mechanisms in which entrepreneurs perceive opportunities and decide to create a company (Baron, 2006; Nixdorff, 2008), leading to considerable interest in the study of entrepreneurial intention (Lee and Wong, 2004; Dimov, 2007b; Liñán and Chen, 2009; Martin et al., 2013; Liñán and Fayolle, 2015). Intentions acting before the start-up of a company are predictors of entrepreneurship conduct (Fayolle et al., 2006), meaning that entrepreneurship intention (starting now EI) could be the first step in forming a company (Lee and Wong, 2004).

The study of EI is the core of various theories and models helping to understand entrepreneurship as a phenomenon that goes beyond the mere creation of companies. An appropriate reference framework for his study has been the Cognitive Social Theory (from now on TSC) (Bandura and Cervone, 1986), which addresses various aspects of the relationship between behavior and intention. Today, a growing body of literature attaches an essential role to EI when deciding to create a company. Thus, several researchers have focused on cognitive aspects to explain and understand the mechanisms through which an individual carries out entrepreneurship behavior (from now on EB), differentiating those with entrepreneurship potential and abilities from the rest (Baron, 1998; Allinson et al., 2000; Sánchez-Almagro, 2003).

Shaver and Scott (1991) and Baron (2004) have highlighted the importance of cognitive variables in understanding these personal decisions. In turn, Robinson et al. (1991) defended the advantages of adopting an approach based on the attitudes of entrepreneurs after finding weaknesses in studies based on personality traits of the entrepreneur. Studies based on the cognitive aspects of individuals explain aspects in which studies based on personality traits showed limitations, providing more robust elements for identifying, measuring, and assessing individuals' potential and entrepreneurship capacities. In this line, the EI has shown to be the most suitable construct in the study of entrepreneurship (Martin et al., 2013).

In this context, various authors have resorted to social psychology in search of a theoretical model that allows explaining the EI from the interaction between personal and social factors. Ajzen's Theory of Planned Behavior (from now on TPB) (Ajzen, 1987, 1991, 2001, 2002) has become the most widely used theoretical framework in EI research (Martin et al., 2013; Fayolle and Liñán, 2014; Liñán and Fayolle, 2015). Under the above arguments, understanding how academic-scientists interpret EI seems to be a central issue in the AE study. It is about these aspects where this research becomes particularly relevant.

### The Chilean Scientific Structure

Chile is one of the leading countries in scientific production in Latin America, being fourth in the total production of SCOPUS indexed publications, under Brazil, Argentina and Mexico (SCImago, 2021), while having a significantly smaller population and a lower GDP in R&D, circa 0.35% (UNESCO, 2021). Astudillo (2014) mentioned several of the shortcomings of the Chilean science policy, highlighting the competitiveness and productivity of the Chilean Scientists in the middle of a least than organized scientific system.

The backbone of the Chilean scientific funding is the National Fund for Scientific and Technological Development or FONDECYT, by its Spanish acronym, a grant given to and mainly administered by a Principal Investigator, who can even change the sponsoring academic institution during its implementation. These grants can have a duration between 2 and 4 years and can be applied mainly to whichever area the researcher wants to research on. FONDECYT is a highly competitive grant, situation that has only been furthered in recent years. One of the reasons for this is Becas Chile, a scholarship program which since 2008 has supported more than about 10.000 doctoral students but which was not followed by a subsequent increase in the number of post-doctoral places or FONDECYT grants to establish these new researchers. Problems with science policy led in late 2015 to strikes and public demonstration by scientists, who took to the streets to complain for the precarious conditions of scientific work in the country. The name of the main organization leading the demonstrations says it all: Science with a contract (Rabesandratana, 2015). Ironically, one of the main reasons for the precarious labor conditions of junior researchers and lab technicians was FONDECYT itself.

### FONDECYT Grants as Start-Up Projects

FONDECYT, comprises three level of grants: post-doctoral, early career, and regular. Each of them has different levels of funding, as well as monetary incentives for the research team. In the post-doctoral grant, most of the grant is put toward the funding of the main researcher, who is an unestablished academic, whereas the early career and regular grant requires the researcher to work on a sponsoring institution on a regular basis, receiving a maximum of USD 700 monthly for their participation on the project. In these two grants, most of the funding goes toward operational expenses, where the Principal Investigator can set up a research team.

The model FONDECYT grants are structured requires skills such as administering funding (up to USD 70.000 per year), setting up a team and lead the implementation of a lab or working installations, while reporting yearly the advance of the project as well as fulfilling the regular activities of an academic post. To successfully establishing themselves as FONDECYT researchers and therefore, advance in their careers, academics in Chile need to learn basically to run a small company, based on the funds obtained from FONDECYT: pay salaries for coinvestigators and research assistants, control the budget, declaring expenses and, also, try to do some science.

One of the limitations that FONDECYT has is related to the expenses it covers, which change every year, and that were at the core of the demonstrations of junior scientists on 2015, because it did not allow to cover social security, holidays o severance payments for them (Rabesandratana, 2015). It also has the limitation that, while for teaching each academic has to go through pedagogical training, basically no one gets trained on how to direct a project. This means that these skills are usually acquired in the most classical way possible: trial and error.

FONDECYT researchers, aside of being the holders of the most prestigious research grant in the Chilean system, become some sort of entrepreneurs, making them ideal to become leaders of start up companies associated to their scientific work. Nonetheless, the tradeoff between administrative tasks derived from the grant administration and having the role of researchers on top of their regular academic obligations makes this group an especially interesting one to assess the factors affecting their Entrepreneurial Intention.

### This Research

This work seeks to answer the following research questions: (1) To what extent do academic-scientists who obtain FONDECYT grants intend to form a company based on the results of their research? (2) To what extent do TPB-based models explain and predict academic-scientific IE? (3) How and to what extent does perceived subjective social support explain and predict the entrepreneurship intention of academic-scientists?

We used a quantitative approach: A survey was implemented in 2017 to the full list of researchers who obtained a FONDECYT grant -in all their forms- between 2007 and 2017. The design is non-experimental, cross sectional and correlational.

## Theoretical Framework

### Entrepreneurial Intention and Behavior

Empirical evidence has enabled, in various studies, to test how TPB-based models explain how many entrepreneurs decide to launch a business, even long before the emergence of an opportunity. Today there is a consensus, based on the results shown by research carried out within the framework of social psychology (Carsrud and Brännback, 2011), that EI is an adequate predictor of the future EB. As the formation of companies is deliberate, intentional, and planned conduct, for many researchers in the field, the EI is the best precedent for predicting it (Bird, 1989; Krueger and Carsrud, 1993).

Coming from psychology, TPB is currently applied in various areas of knowledge, such as marketing (Sheppard et al., 1988) or career choice (Ajzen, 2001). In the specific field of entrepreneurship, EI is explained based on three dimensions: (1) Attitude toward Entrepreneurship (AT); (2) Perceived subjective social support and (SN); (3) Perceived Behavioral Control (CN) (Kolvereid, 1996).

Ajzen (1991) notes that these dimensions can have different weights as predictors of EI, depending on the behavior analyzed, contexts, and situations in which the individual operates. Research shows that these variables explain between 30 and 45% of the variance of EI in an individual (Kolvereid, 1996; Krueger et al., 2000; Autio et al., 2001; Liñán and Chen, 2009).

Engle et al. (2010) researched 12 countries, confirming that TPB significantly predicts EI, showing different results at the country level. The percentage of variance explained from the EI ranged between 9 and 42%. These results support the importance of context proposed by Ajzen (1991).

Research has made it possible to distinguish two categories of factors that have effects on EI: (1) Individual-level and; (2) Context or Environmental level. Sarason et al. (2006) and Dimov (2007a) propose that EI is a construct that operates as a mediator between the conception of an idea and its subsequent transformation into entrepreneurial action. Some authors also propose that, in the case of an entrepreneurial idea, the EI also involves collecting data and information, allowing the individual to assess the feasibility of a given entrepreneurship idea properly (Dimov, 2007b; Hayton and Cholakova, 2012).

Evidence to date shows the minimal difference between various approaches applied in behavioral prediction, such as EI models (Krueger, 1993, 2000; Shapero and Sokol, 2002) and the TPB (Krueger et al., 2000). Research has also consistently shown the predictive capacity of the EI over the EB (Kuehn, 2008; Liñán, 2008; Elfving et al., 2009; Iakovleva et al., 2011; Kautonen et al., 2011), further proving that the AT and CN have direct effects on the EI (Wood and Bandura, 1989; Bandura, 1997). In turn, other investigations have not consistently verified the effect of SN on EI (Carsrud and Brännback, 2011). Finally, various studies (Kolvereid, 1996; Tkachev and Kolvereid, 1999; Kautonen et al., 2013), conducted on different populations, show that the three factors have significant effects on EI.

To date, there is little research in the area of the AE that addresses the study of the EI-EB relationship, focusing on scientists as a relevant actor of this phenomenon (Jain et al., 2009). This lack of research is evident even when they have the potential of founding science-based startups.

One of the studies focusing on this population (engineering professors) shows that perceptions of uselessness and lack of feasibility of assuming entrepreneurship behavior affect the EI of academics (Llano, 2010). Recent research has tried to fill this gap (Kolvereid and Isaksen, 2006; Kautonen et al., 2013; Gielnik et al., 2014; Van Gelderen et al., 2015), showing each of these studies, the predictive power of EI on EB. However, research giving attention specifically to AE is scarce. One of these investigations, on a German academic-scientist population, is carried out by Goethner et al. (2012). They confirm with their results that EI predicts the subsequent formation of an academic spin-off. Llano (2010), conducting a study on a population of researchers from different U.S. universities, verifies that the EI predicts acceptably several EB, such as obtaining patents, the formalization of licensing contracts of the same, and the formation of academic spin-offs by these researchers. Finally, Standish (2007) finds that the intention of researchers from New York universities to exploit the results of their research adequately commercially predicts their subsequent commercial behavior.

### Entrepreneurial Intention and Entrepreneurial Attitude

Attitude toward Entrepreneurship is the desire of an individual to carry out a specific entrepreneurship behavior to create value (Fini et al., 2012). The predictive quality of AT on EI and its effect on future EB has been verified in several research works, empirically proving the relationship between these constructs (Iakovleva et al., 2011; Fini et al., 2012; Moriano et al., 2012).

The results obtained from several meta-analyses on TPB have provided empirical evidence regarding the direct relationship between attitude and intention, verifying that attitude operates as a consistent and robust predictor of intention (Albarracín et al., 2001; Hagger et al., 2002; Cooke and Sheeran, 2004; Arvola et al., 2008).

The claim that attitude has an indirect effect on behavior, mediated by intention, has also been supported. Ajzen and Fishbein (2005) proposed that measuring the attitude toward a particular behavior, and not toward an object, would allow a better analysis of the attitude-behavior relationship.

In simple terms, the more positive the attitude toward a particular behavior, the greater the intention to carry it out (Armitage and Conner, 2001). Since the attributes associated with a given behavior are valued positively or negatively by the individual, this assessment will form the individual's attitude toward such conduct. An individual will create a positive attitude toward conduct which, in his view, gives him favorable consequences and, on the contrary, He will develop a negative attitude toward behavior which, in his view, gives him unfavorable outcomes (Ajzen, 1991).

Attitudes include both the psychological assessment toward a specific behavior (Ajzen and Fishbein, 2005) and the intensity of this assessment, reflecting the importance that the individual attaches to him (Klein and Sorra, 1996).

Although attitude theories adopt among their central assumptions that individuals' assessments of a specific object are stable over time, research shows that people can present various attitudes toward a given object (Wood, 2000). Also, individuals can display two parallel attitudes, even within the same context (Wilson et al., 2000). Also, individuals themselves may present different attitudes toward an object, depending on the context in which it is located (McConnell et al., 1997).

Research on AE has mainly adopted the theoretical framework proposed by Ajzen (1991), where attitudes express the permanent valuation, either positive or negative, of carrying out a specific behavior (Goethner et al., 2009, 2011, 2012). Research has also found a tension between academic values and the commercial exploitation of research (Martinelli et al., 2008) and that academics-scientists are reluctant to carry out entrepreneurial behaviors (Bird and Allen, 1989).

Other authors suggest that some academic-scientists have positive attitudes that make them more likely to exploit the results of their research commercially, or that they have previous skills or knowledge that gives them a greater capacity to recognize entrepreneurial opportunities (Etzkowitz, 1983; Shane, 2000; Azoulay et al., 2007). Academic-scientists who positively value their entrepreneurial behavior based on the commercial exploitation of the results of their research, a positive disposition is verified in favor of investing resources, time, and effort to materialize an entrepreneurial behavior (Gulbrandsen, 2005; Goethner et al., 2009).

The AT, called “Attitude to Conduct” in the context of TPB, is the most critical proximal cognitive factor of EI, taking into account its direct effect on it and its indirect effect on the EB (Bagozzi, 1992; Ajzen and Fishbein, 2005). The attitude can be conceived as learned and implicit responses of varying intensity, operating as a mediator or guideline in the process of valuation toward a specific object or concept made by an individual (Fishbein and Ajzen, 2010).

Considering this, the first hypothesis we propose is:

- H1: Attitude toward academic entrepreneurship has a direct, positive, and significant effect on the entrepreneurship intention of academic scientists.

### Entrepreneurship Intention and Perceived Behavioral Control

Perceptions are structural components of TPB and TSC. More specifically, perception of control has emerged as an essential concept in behavioral research, both theoretically and empirically (Ajzen, 2002; Trafimow et al., 2002; Kraft et al., 2005). Previous research has shown that constructs associated with perceptions of control operate as valuable indicators, either of behavior and the social functions of individuals (Skinner, 1996).

Ajzen (1991) included the CN construct to account for conducts over which subjects do not exercise entirely voluntary control. Since many behaviors do not fully respond to motivation but require the perception of some form of self-skill, the CN is associated with positive self-assessment, concerning one's abilities and abilities to perform specific behavior in certain situations and contexts (Ajzen, 1991).

CN is the difficulty or ease perceived by an individual to carry out a specific behavior. It is determined by beliefs of control, which relate, in turn, to the opportunities and resources available, necessary to materialize the action and, at the same time, to the assessment of the capacity that these opportunities and resources have to block or facilitate the behavior (Ajzen, 1991).

Control beliefs may be rooted in subjects, given their previous experience concerning certain specific behaviors. It is also influenced by secondary information from other individuals regarding that behavior, whether by the experience of friends, family, colleagues, or other factors that reduce or increase the perceived difficulty or ease of carrying out the conduct in question. In this way, the greater the support that individuals have and the fewer barriers or impediments they see, the greater their CN (Ajzen, 1991).

The relationship between conduct and CN is explained by two mechanisms (Ajzen, 1985): (1) When intention does not vary, an individual's effort to perform a specific behavior will increase when the individual perceives that he or she has greater control and therefore perceives a greater likelihood of success until positive results are achieved; (2) The CN can serve as a real control measure to predict the probability of success in the performance of a behavior (Herrero Crespo, 2005).

In TPB, CN has a double effect on behavior. On the one hand, it has a direct effect and, on the other hand, an indirect effect mediated by intention. There is a direct effect of the CN on behavior when perceived control acts as a substitute for current control, which depends heavily on the realism of perceptions. When there is insufficient information, or the individual encounters unusual or unknown situations, there could be a misperception about his or her abilities and the skills necessary to perform the behavior. In this scenario, the CN does not operate as an accurate control measure and loses its predictive capacity over behavior (Ajzen and Madden, 1986; Ajzen, 1991). On the other hand, there is an indirect effect when the individual believes he has the necessary skills and abilities to carry out behavior and is willing to make a more significant effort to materialize it.

From the perspective of psychology, a key issue is the perception of control over behavior and how this affects intentions and subsequent behavior (Ajzen, 1991). Individuals may find themselves, in certain situations, conditioned by non-motivational factors related to the availability of opportunities, skills, capabilities, and resources, limiting their decision-making power when assuming a given behavior (Rosenstock, 2005). To account for this literature, we will test the following hypothesis:

- H2: Perceived behavioral control toward academic entrepreneurship has a direct, positive, and significant effect on the entrepreneurial intention of academics-scientists.

### Entrepreneurial Intention and Perceived Subjective Social Support

The Subjective Norms construct may be contradictory. They are understood as subjective given their inner nature: it implies that the individual carries out a decision process by evaluating his reference groups' opinion concerning his behavior by himself. They are understood as “norms” since the individual, depending on the evaluation he makes, adopts them as behavioral guidelines (Fini et al., 2012). SN are conceived within the framework of the TPB as the pressure and/or social support that an individual perceives when deciding to carry out a specific behavior (Fini et al., 2012). In both theories, the influence, support, or social pressure that the individual perceives is represented by the SN construct. This cognitive factor, which is proximate to EI, expresses the individual's perception regarding the approval, or not, of his or her reference groups when faced with the decision to carry out a particular behavior (Ajzen, 1991). The reference groups refer to people against whom the individual is compared (Herrero Crespo, 2005). These reference groups could be family members, co-workers, and close friends.

The importance of the SN has been recognized for its impact on behavior (White et al., 2009). In the specific study of the enterprise, this construct has shown a weak explanatory force of the EI, having rather indirect effects on it, through the AT and CN (Armitage and Conner, 1999, 2001; Krueger et al., 2000; Liñán and Chen, 2009; White et al., 2009; Alonso, 2012; Liñán et al., 2013; Hui-Chen et al., 2014). This indirect relationship is reported in studies that incorporate social capital as a distal construct (Liñán, 2008; Liñán et al., 2011), differentiating the effects of the SN on the EI, depending on whether the subjects have a strong action orientation (Bagozzi, 1992) or if they present a strong locus of control (Ajzen, 2002).

Some literature on entrepreneurship reports a positive and direct relationship between the SN and the EI (Kolvereid and Isaksen, 2006; Carr and Sequeira, 2007; Engle et al., 2010). Other authors report a weak effect of the SN on the EI (Fini et al., 2012), with significant variations depending on the type of population and culture under study (Moriano et al., 2012). These results mean that there are differences in the predictive power of the models applied that could be due to cultural factors. Some researchers have even proposed replacing this construct with others, such as social influence (White et al., 2009) or self-identity (Sparks and Shepherd, 1992; Armitage and Conner, 1999), arguing that this could improve the predictive power of the models.

In any case, most individuals' central reference group is their family, as it provides the fundamental value structure, beliefs, norms, and attitudes (Herrero Crespo, 2005). Furthermore, the SN does not operate in the individual because of external social stimuli, such as rewards or punishments, but works on its internal control (Fini et al., 2012), meaning that communication processes are central for their formation, modification, and spreading (Lapinski and Rimal, 2005).

Llano (2010), when conducting a study on populations of academics-scientists from different U.S. universities, supports that the SN does not present significant effects on the EI. On the opposite, Gallurt Plá (2010) finds, in a study conducted on populations of academic-scientists from two Spanish universities, that the SN is the construct that presents the most significant effect on EI.

SN perceived by academic scientists depends strongly on their normative beliefs. Organizational cultures can affect these normative beliefs, for example, depending on how important the organization's entrepreneurial orientation is. In this sense, the SN of an academic scientist regarding the EB could associate with the normative expectations of their peers (Llano, 2010).

Academic scientists with colleagues who are at similar levels of career development and who have carried out entrepreneurial initiatives at the university will have a greater propensity to carry out an EB (Bercovitz and Feldman, 2008). Accordingly, academic scientists in organizational contexts with a clear entrepreneurial orientation, enabling an EB to materialize, should be more likely to engage in entrepreneurial initiatives (Stuart and Ding, 2006).

Although studies of EI are available today in academic scientists are limited, the results until now report a weak effect of SN on EI (Goethner et al., 2009, 2012). These results could be due to the particular and specific characteristics of individuals belonging to this group (highly educated and highly specialized). At the same time, their own beliefs could significantly mitigate the effect of SN on their EI (Ajzen, 1991).

Considering these limitations, we will test the following hypotheses empirically:

- H3: The perceived subjective social norm toward academic entrepreneurship has a direct, positive, and significant effect on the entrepreneurial intention of academics-scientists.
- H4: The perceived subjective social norm toward academic entrepreneurship has a direct, positive, and significant effect on the entrepreneurship attitude of academics-scientists.
- H5: The perceived subjective social norm for academic entrepreneurship has a direct, positive, and significant effect on the perceived behavioral control of academic-scientist entrepreneurship
- H6: The perceived subjective social norm for academic entrepreneurship has a positive and significant indirect effect on the entrepreneurship intention of academics-scientists, mediated by the attitude toward entrepreneurship
- H7: The perceived subjective social norm for academic entrepreneurship has a positive and significant indirect effect on the entrepreneurship intention of academics-scientists, mediated by perceived behavioral control of entrepreneurship.

Table 1 summarizes the hypotheses formulated in this study.

**Table 1:** Hypotheses.

**No**.	**Descriptions**
1	Attitude toward academic entrepreneurship has a direct, positive and significant effect on the entrepreneurship intention of academic-scientists.
2	Perceived behavioral control toward academic entrepreneurship has a direct, positive and significant effect on the entrepreneurial intention of academics-scientists.
3	Perceived subjective social norm toward academic entrepreneurship has a direct, positive and significant effect on the entrepreneurial intention of academics-scientists.
4	Perceived subjective social norm toward academic entrepreneurship has a direct, positive, and significant effect on the entrepreneurship attitude of academics-scientists.
5	Perceived subjective social norm for academic entrepreneurship has a direct, positive, and significant effect on the perceived behavioral control of academic-scientist entrepreneurship.
6	Perceived subjective social norm for academic entrepreneurship has a positive and significant indirect effect on the entrepreneurship intention of academics-scientists, mediated by the attitude toward entrepreneurship.
7	Perceived subjective social norm for academic entrepreneurship has a positive and significant indirect effect on the entrepreneurship intention of academics-scientists, mediated by perceived behavioral control of entrepreneurship.

**Figure 1:**
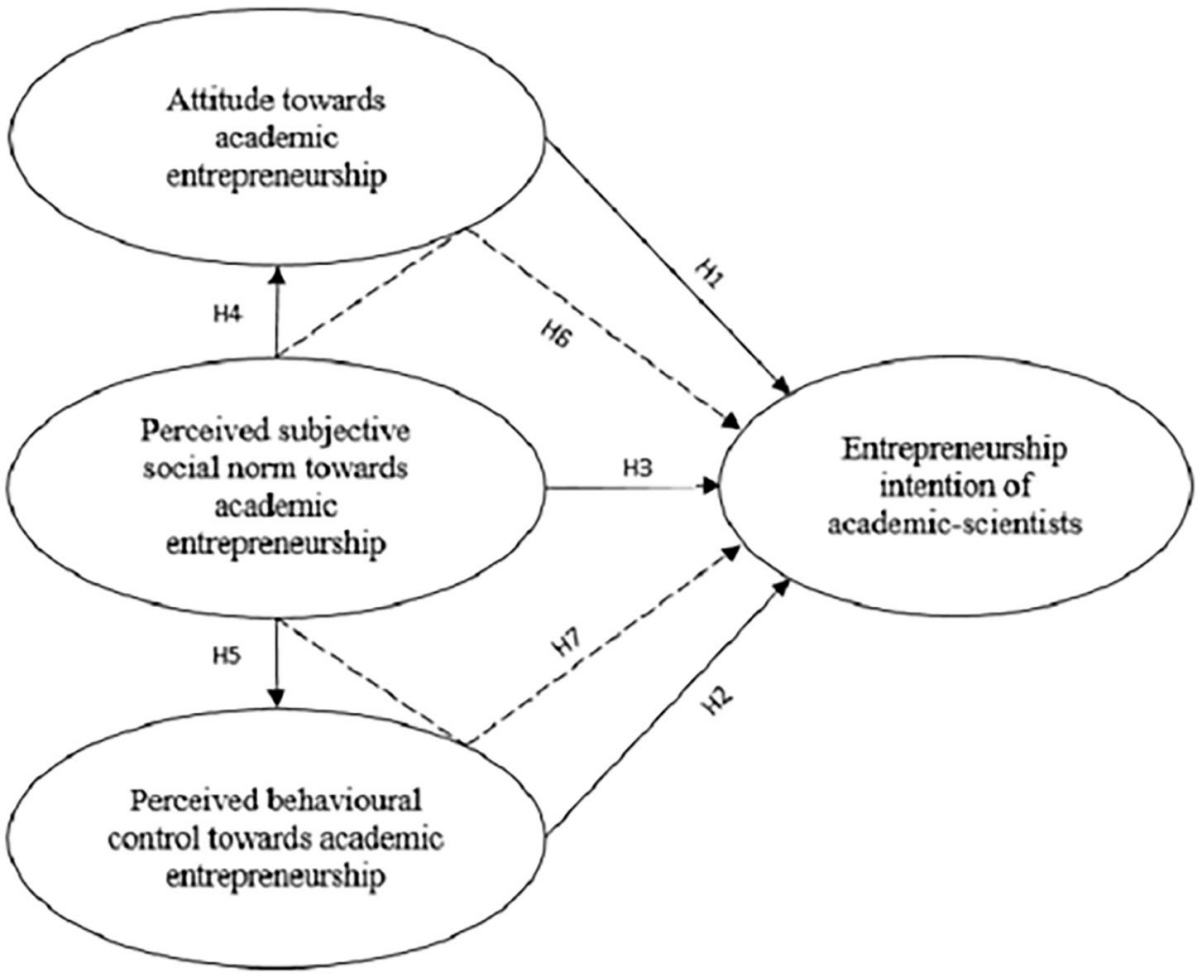
Hypotheses.

Figure 1 presents the proposed relationships and summarizes the primary model of this study. Following Liñán and Chen (2009), the influence of SN on CA and CN will be tested as a model of the relationship between motivational background and EI.

## Methods

### Sample

The sampling framework for this study was composed of all the scientists obtaining a FONDECYT grant between 2007 and 2017. The data for the construction of the sampling framework was publicly available at the website of FONDECYT, where the names and institutions of the grant winners are published. The sampling framework was composed of 5,571 individual academics whose emails were collected via their institutional websites.

Between June 15 and July 16, 2017, a self-reported web survey was sent to the total dataset of 5,571 academic-scientists through SurveyMonkey. A total of 1,027 scientists (18.4%) responded fully. These 1,027 cases were included in the final database, with complete answers in the four scales included in this research.

The survey was reviewed by the ethics committee of the Faculty of Administration and Economics of the University of Santiago de Chile.

For the analysis we used SPSS version 21 and MplusV7.1.

### Questionnaire

The questionnaire included an informed consent and questions related to the demographic profile (age, gender, and nationality); education and employment (higher academic degree, participation in associative research groups, academic rank, type of employment, area of study, and university where they work). Finally, it included variables associated with the entrepreneurial activity (entrepreneurial experience, formation of spin-offs, application for patents, and previous business failures).

### Design and Psychometric Properties of the Measurement Scales

We used the scales developed by Liñán and Chen (2009). The questionnaire was translated into Spanish and validated both with experts and with a pretest. The measurement of the indicators for the four constructs of the Intention model was through Likert-type scales, with sentences expressed in the form of pure intention, evaluating the degree of agreement of the subjects with such sentences (see Appendix). To ensure the comparability of this study and the equivalence of the scales, we followed the methodology set forth by Liñán and Chen (2009), complementing this analysis with other methods suggested in the literature (Muthén and Muthén, 1998–2017; Wang and Wang, 2012).

Discriminant validity of the scales was evaluated based on examining the correlations, adopting as a criterion that each item should correlate more strongly with its construct than with any other. Higher correlations indicate that the respondents perceive each indicator as belonging to their respective theoretical construct rather than another (Messick, 1988). For such purposes, the item-construct correlation was calculated for each factor. As shown in Table 2, correlations of each item with the other constructs are always lower than the average correlation with its construct.

**Table 2:** Item-construct correlation.

**Item**	**Factor**			
	**EI**	**CN**	**SN**	**AT**
IE_4	0.90	0.66	0.54	0.79
IE_3		0.67	0.53	0.79
IE_5		0.67	0.54	0.77
IE_1		0.63	0.51	0.76
IE_2		0.62	0.48	0.82
IE_6		0.56	0.40	0.70
CN_3	0.63	0.85	0.52	0.59
CN_2	0.67		0.50	0.62
CN_6	0.57		0.51	0.54
CN_4	0.58		0.37	0.46
CN_5	0.55		0.36	0.43
CN_1	0.58		0.51	0.58
NS_1	0.48	0.47	0.88	0.60
NS_2	0.51	0.49		0.61
NS_3	0.46	0.46		0.57
AC_4	0.77	0.57	0.62	0.88
AC_2	0.78	0.60	0.61	
AC_3	0.78	0.59	0.64	
AC_5	0.75	0.56	0.58	
AC_1	0.68	0.57	0.58	
IE	–	0.67	0.51	0.81
CN	0.67	–	0.50	0.59
NS	0.51	0.50	–	0.63
AC	0.81	0.59	0.63	–

*EI: Entrepreneurship Intention; AT: Attitude toward Entrepreneurship; SN: Perceived subjective social support toward Entrepreneurship; CN: Perceived Behavioral Control toward Entrepreneurship*.

After checking the validity analysis of the scales, we used Exploratory Factor Analysis to identify, for each scale, the most appropriate indicators. Kolmogorov-Smirnov test did not support the normality of the items' distribution. Because of this finding, the extraction method selected for EFA was the factorization of main axes, imposing the extraction of 4 factors. The accumulated variance explained by the four factors reached a total of 78.8%. Table 3 presents the original Cronbach's Alpha for the four scales, with values of α between 0.911 and 0.966.

**Table 3:** Rotated factor matrix and reliability indicators.

**Item**	**Factor**			
	**EI**	**CN**	**SN**	**AT**
IE_4	0.92			
IE_3	0.90			
IE_5	0.89			
IE_1	0.86			
IE_2	0.62			
IE_6	0.52			
CN_3		0.88		
CN_2		0.86		
CN_6		0.84		
CN_4		0.82		
CN_5		0.77		
CN_1		0.69		
NS_1			1.02	
NS_2			0.84	
NS_3			0.67	
AC_4				−0.86
AC_2				−0.85
AC_3				−0.66
AC_5				−0.62
AC_1				−0.58
Cronbach's α	0.966	0.941	0.911	0.947

*Extraction method: Principal axis factorization. Rotation method: Oblimin Normalized with Kaiser. Rotation converged after 14 iterations. EI: Entrepreneurship Intention; AT: Attitude toward Entrepreneurship; SN: Perceived subjective social support toward Entrepreneurship; CN: Perceived Behavioral Control toward Entrepreneurship*.

Finally, the original versions of the scales were adjusted to increase alpha. Table 4 presents the final version of the scales.

**Table 4:** Modified rotated factor matrix and reliability indicators.

**Item**	**Factor**			
	**EI**	**CN**	**SN**	**AT**
IE_4	0.93			
IE_1	0.91			
IE_5	0.88			
IE_3	0.87			
CN_4		0.88		
CN_5		0.86		
CN_6		0.83		
CN_3		0.82		
CN_2		0.76		
CN_1		0.69		
NS_2			1.05	
NS_1			0.85	
NS_3			0.68	
AC_4				−0.98
AC_2				−0.80
AC_5				−0.70
AC_3				−0.65
Cronbach's α	0.973	0.941	0.911	0.948

*Extraction method: Principal axis factorization. Rotation method: Oblimin Normalized with Kaiser. Rotation converged after 14 iterations. EI: Entrepreneurship Intention; AT: Attitude toward Entrepreneurship; SN: Perceived subjective social support toward Entrepreneurship; CN: Perceived Behavioral Control toward Entrepreneurship*.

## Results

### Demographics

The sample is composed of 322 women (31.4%) and 705 men (68.6%). Six hundred nine cases (59.3%) are in the range of 30–59 years, while 4 cases (0.4%) are between 20 and 29 years, 81 cases (7.9%) are 60 years or older, and 333 cases (32.4%) did not declare their age.

Women concentrate mainly in the range of 30–39 years (87 cases, 8.5%), followed by the range of 40–49 years with 81 cases (7.9%) and then 35 cases (3.4%) in the range of 50–59 years. Men are mainly in the range of 40–49 years (177 cases, 17.3%), followed by the range of 30–39 years with 144 cases (14.1%) and then 83 cases (8.1%) in the range of 50–59 years.

Concerning nationality, the sample is composed of 652 Chileans (63.5%), 94 foreign nationals (9.2%), and 281 cases without nationality classification (27.4%). Chilean researchers are found mainly between 40 and 49 years (219 cases, 21.3%), followed by the range of 30–39 years with 195 cases (19.0%) and then 106 cases (10.3%) in the range of 50–59 years. Foreign researchers follow a similar pattern, with most found in the range of 40–49 years, 38 cases (3.7%), followed by the range of 30–39 years with 37 cases (3.6%) and then 12 cases (1.2%) in the range of 50–59 years.

Regarding the type of FONDECYT grant, 529 cases (51.5%) are recipients of the Regular fund, 374 cases (36.4%) of the early career fund, and 124 cases (12.1%) of the Post-doctoral fund.

The area of research that presents more responses is engineering (186; 18.1%), followed by biology (157; 15.3%), chemistry (96; 9.3%), medicine (90; 8.8%), agronomy (67; 6.5%), and mathematics (44; 4.3%). These six areas account for 58% of the answers obtained. The above is consistent with the proportion of all FONDECYT researchers distributed by area of study.

Concerning the configuration of the researchers' work profile, 363 cases (35.3%) declared to participate in associative research teams, 374 cases (36.4%) declared not to participate, while 290 cases (28.2%) did not respond. The highest concentration by age range is between 30 and 49 years old, with 491 cases (47.8%) and 50–to 59 years old with 118 cases (11.5%). For their part, 680 cases (66.2%) are in full-time mode, 45 cases (4.4%) in part-time mode, while 302 cases (29.4%) could not be classified.

As for the entrepreneurial profile of the researchers surveyed, 225 researchers (21.9%) had applied for a patent. Ninety-four researchers (9.2%), had been granted a patent, 163 (15.9%) had formed a company, 39 (3.8%) had developed a spin-off, and 62 (6.0%) declared a previous business failure. According to these indicators, the Regular (senior) fund researchers show higher entrepreneurial activity compared to the researchers of Initiation and Post-doctoral.

#### Entrepreneurship Intention Measurement Model

**Figure 2:**
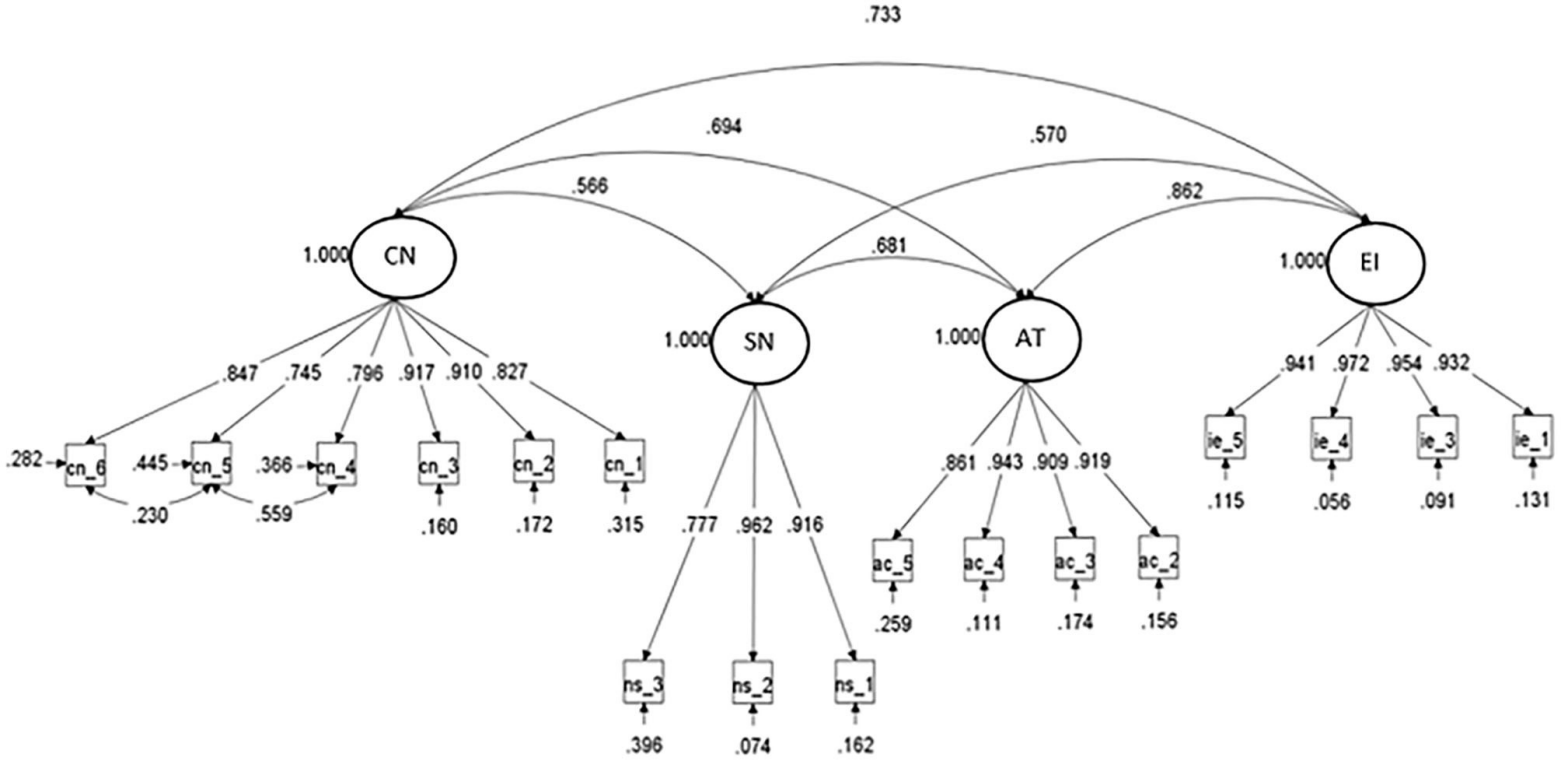
Entrepreneurship intention—measure model.

To assess the joint reliability of the scales, and therefore the feasibility of a complete model, we used SEM confirmatory factor analysis (CFA-SEM). This analysis allows estimating, on the one hand, the composite reliability index of the scales and, on the other hand, the goodness-of-fit indexes of the measurement model (Figure 2), as proposed by Wang and Wang (2012).

As mentioned previously, the sample data does not follow a normal distribution. The estimation method applied was Maximum Robust Likelihood to Non-Normality of Variables (MLR; Muthén and Muthén, 1998–2017). The composite reliability indices present good values as recommended (ρ between 0.884 and 0.973), above the recommended minimum (ρ > 0.7), as shown in Table 5.

**Table 5:** Composite reliability index.

**Item**	**Factor**			
	**EI**	**CN**	**SN**	**AT**
IE_1	0.932			
IE_3	0.954			
IE_4	0.972			
IE_5	0.941			
AC_2		0.919		
AC_3		0.909		
AC_4		0.943		
AC_5		0.861		
CN_1			0.827	
CN_2			0.910	
CN_3			0.917	
CN_4			0.796	
CN_5			0.745	
CN_6			0.847	
NS_1				0.916
NS_2				0.962
NS_3				0.777
Composite reliability index	0.973	0.884	0.918	0.950

*EI: Entrepreneurship Intention; AT: Attitude toward Entrepreneurship; SN: Perceived subjective social support toward Entrepreneurship; CN: Perceived Behavioral Control toward Entrepreneurship*.

The model's goodness of fit indexes are above the suggested minimum values (χ^2^ = 316,996; *df* = 111; *p* < 0.001; RMSEA = 0.043, IC90%: 0.037–0.048; CFI = 0.984; TLI = 0.980; SRMR = 0.027) for this type of analysis (Wang and Wang, 2012). With respect to the measurement model, only the standardized factorial loads that were significant are shown in Figure 2.

#### Structural Model of Entrepreneurship Intention

**Figure 3:**
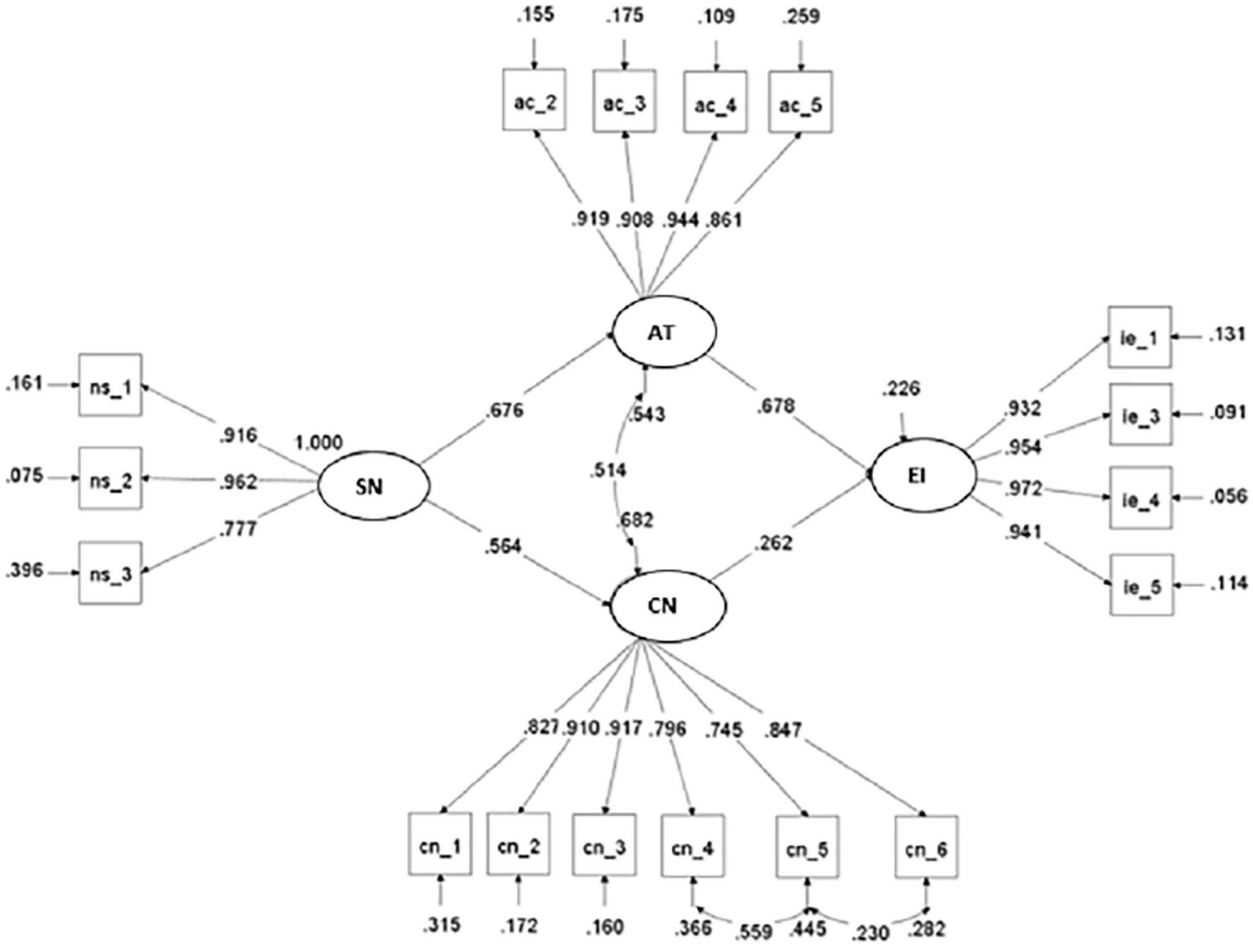
Entrepreneurship intention—structural model.

We used SEM with Maximum Robust Likelihood to check our hypotheses. The model tested is presented in Figure 3. The indexes of the goodness of fit are above the suggested minimum values for this type of analysis (Muthén and Muthén, 1998–2017; Wang and Wang, 2012) (χ^2^ = 325,773; *df* = 112; *p* < 0.001; RMSEA = 0.043, IC90%: 0.038–0.049; CFI = 0.983; TLI = 0.979; SRMR = 0.027).

In Figure 3, the basic model of entrepreneurial intent of academics-scientists is supported, except in SN → EI's relationship. Up to this point, hypotheses H1, H2, H4, and H5 have been supported, but not hypothesis H3.

We can see that AT and CN present a direct, positive, and significant effect (*p* < 0.0001) on EI and, at the same time, SN shows a direct, positive, and significant effect (*p* < 0.0001) on both AT and CN. Nonetheless, the results could not prove a direct, positive, and significant (*p* > 0.05) effect of SN on EI.

Similarly, when testing the H6 and H7 hypotheses, the results of the indirect model show that SN has a total indirect, positive, and significant effect (*p* < 0.001) on EI (NSIND → EI = 0.606). More specifically, it has an indirect, positive, and significant effect (*p* < 0.0001) through AT (0.458) and an indirect, positive, and significant effect (*p* < 0.0001) through CN (0.148).

These results are consistent with previous studies, where SN affects EI, through its effect on AT and CN. These results show that the hypotheses H6 and H7 find empirical support in the sample data. Table 6 reports the empirical testing of the hypotheses, while Table 7 reports the direct and indirect effects of the variables of the base model on EI.

**Table 6:** Results of the empirical support of the hypotheses.

**Hypotheses No**.	**Relationship**	**Support**
1	AT –> EI	Support
2	CN –> EI	Support
3	SN –> EI	No support
4	SN –> AT	Support
5	SN –> CN	Support
6	SN –> AT –> EI	Support
7	SN –> CN –> EI	Support

**Table 7:** Direct and indirect effects.

**Factor**	**Total**	**Total Indirect**	**Specific Indirect**		**Direct**
			**SN → AT → EI**	**SN → CN → EI**	
AT	0.678^**^				0.678^**^
CN	0.262^**^				0.262^**^
SN	0.606^**^	0.606^**^	0.458^**^	0.148^**^	

*Only significant path coefficients included*.

** *p < 0.001*.

The report of effect sizes based on R2 shows that the model explains 77.4% of EI variance and that AT has a larger effect than CN, which is established in the values of the standardized regression coefficients, where λAT → EI = 0.678 and λCN → EI = 0.262. It also shows the contribution of SN in explaining 45.7 and 31.8% of the variance in AT and CN, respectively.

These results can be considered satisfactory compared to values reported in previous studies. Most previous research used multiple linear regression models, which have managed to explain around 40% of EI variance (Liñán and Chen, 2009; Liñán and Fayolle, 2015).

## Discussion

The findings presented in this article show empirical support for the proposed model of entrepreneurial intent of academics-scientists. This work joins a body of research using TBP as a fruitful theoretical framework for the study of intentions, particularly EI.

The SEM analysis presented a significant indirect role of Perceived Social Support on Entrepreneurial Attitudes and Perceived Behavioral Control. Many previous studies have discarded the SN from the model based on multiple linear regressions because it does not show a direct relationship to the Entrepreneurial intention. This work makes clear the need not to neglect its effects, direct, and indirect.

In the case of FONDECYT holders, we can say that entrepreneurial intention answers to the same structure of relationships of its predictors: attitude and control. They are affected by perceived social support, which indirectly affects entrepreneurial intention.

Based on the available literature, it is possible to say that variations in the intensity of these relationships could depend on different perceptions, given cultural or contextual differences (Liñán and Chen, 2009).

Nonetheless, considering the characteristics of the population under study: elite researchers with a nationally prestigious research grant, who, as part of this grant, have to form and manage teams, these results are interesting for two main reasons:

The first is that elite researchers are high achievers, meaning that we analyze individuals with high self-efficacy and a proven track record. Therefore, these results, showing that the perception of social support would influence their levels of entrepreneurial attitude and behavior control, having a positive indirect effect on their intention of creating science-based companies, is relevant. Even when results could be similar to other social groups, this does not have the same implications for elite scientists: They can create new companies.

These results highlight a new path for scientific policy in highlighting the indirect effects of perceived social norms on the entrepreneurial intention of elite scientists. Can academic entrepreneurship be promoted fruitfully as an individual endeavor, or is it necessary to encourage entrepreneurship as part of an organizational change? Our results suggest that without changing the social norms behind academia, it could be harder to promote changes in attitudes and perceived controllability, facilitating entrepreneurial intention.

The case of Chile is unusual in Latin America in terms that its universities are identified as more entrepreneurial and have a more individual model of research funding through FONDECYT. It has also seen a growth in private research universities, which have tried to develop an entrepreneurial focus, meaning a switch from the institutional model prevailing in the region (Bernasconi, 2005; Pineda, 2015). One of the main strategic challenges for the universities to become more entrepreneurial is to develop entrepreneurial abilities among their members (Klofsten et al., 2019) while at the same time avoiding making them fall under an indicators-based system of academic administration, which could limit their capacity to produce new ideas (Fardella et al., 2019, 2020) and undermine their job and life satisfaction (Unanue et al., 2017).

Finally, there is a space for the most relevant human reference group: families. Research is a demanding profession, where significant hours are put toward the romantic notion of advancing human knowledge, aside from those devoted to teaching and administrative responsibilities associated with an academic position. These demands constantly mean a work-family conflict that is not solved until the reach of job stability and promotion, reserved for researchers with a track record of publications and grants. This conflict is even challenging for women (Fardella and Corvalán, 2020). Our results show that promoting entrepreneurial intention should not be limited to the university space. It necessarily has also to reach researchers' families. If we do not consider this trade-off between work and family when promoting entrepreneurial intention, we will not facilitate its acceptability. Instead, we will make this conflict evident, alienating researchers from taking risks associated with business creation.

These results support the idea that policies orientated to the promotion of academic entrepreneurship should consider, among their components, the creation of a supportive environment toward the creation of companies arising from research results and that that environment includes the researchers' reference groups. In this sense, communication strategies focusing on promoting entrepreneurship should understand the social norms guiding the target groups' actions to be effective.

## Limitations and Possible Ways Out

The primary limitations found in this work are two-fold: firstly, methodological: its cross-sectional design limits the possibility of measuring the effect of these constructs on entrepreneurial behavior. We expect further research to address this issue. Secondly, theoretically it seems necessary to further research on the predicting role of perceived social support on entrepreneurial intention and behavior. It seems particularly important to disentangle the role of perceived organizational and familial support on entrepreneurial intention and, particularly, on entrepreneurial behavior. It is also necessary to keep working on how institutional support is perceived differently across disciplines, while in business and engineering the creation of companies seemed natural, in humanities it was, until recently with the development of the digital humanities, a stranger path to follow.

Elite scientists, such as FONDECYT holders, are among the most prepared and capable people a country has. Promoting entrepreneurship among them is a critical factor for creating science-based companies, which could successfully address societal challenges. Understanding the specific needs of this group for initiating a company is a crucial factor in creating value in developing economies.

## Data Availability Statement

The raw data supporting the conclusions of this article will be made available by the authors, without undue reservation.

## Ethics Statement

The studies involving human participants were reviewed and approved by Ethics Committee of the Faculty of Administration and Economics of the Universidad de Santiago de Chile. The patients/participants provided their written informed consent to participate in this study.

## Author Contributions

EA-D conceptualized the study, chose the theoretical framework, and measures. EA-D and JO designed the general study and the methods to be implemented. DP-W and RJ-G contributed to the literature review. All authors listed have made substantial, direct and intellectual contribution to the work, wrote several sections of the initial draft, carried out analysis and interpreted results, wrote, read, and revised the final paper and approved it for publication.

## Conflict of Interest

The authors declare that the research was conducted in the absence of any commercial or financial relationships that could be construed as a potential conflict of interest.
