# Supplementary figures and images for: Entrepreneurial Intention and Perceived Social Support From Academics-Scientists at Chilean Universities
Source: Front Psychol. 2021 Jul 7;12:682632. doi: 10.3389/fpsyg.2021.682632 (PMC8292899; doi:10.3389/fpsyg.2021.682632)

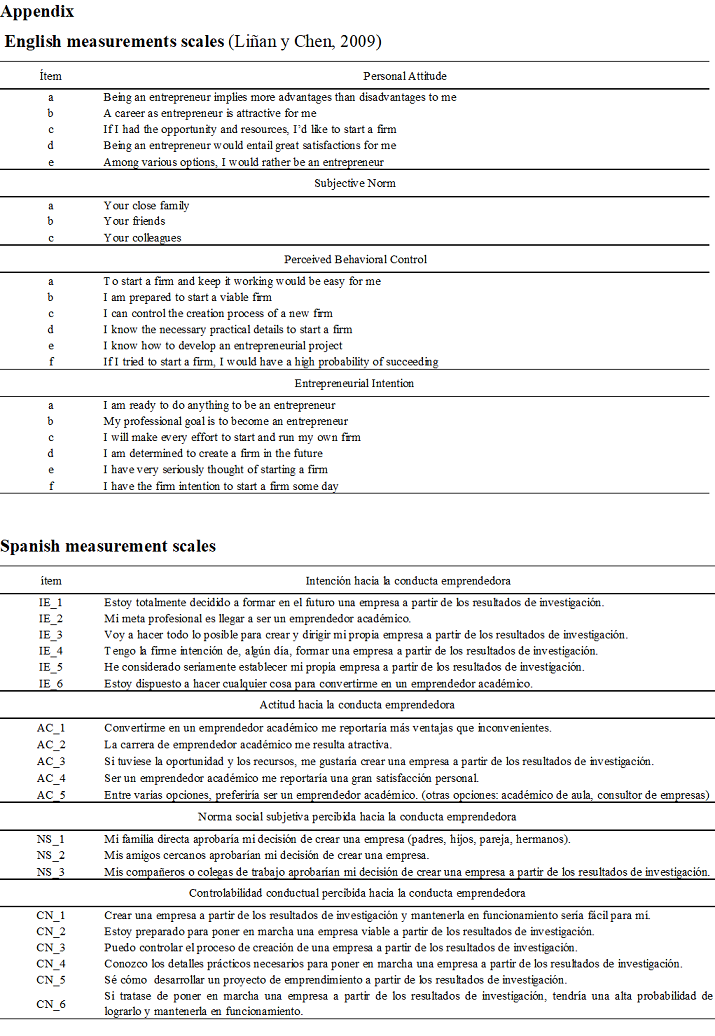

Supplement: Supplementary file 1 [file Image_1.TIF]
